# Malignant lymphoma of mucosa-associated lymphoid tissue involving the renal pelvis and the entire ureter: A case report

**DOI:** 10.3892/ol.2013.1221

**Published:** 2013-03-01

**Authors:** HIDEO OTSUKI, KEIICHI ITO, KEN SATO, TAKEO KOSAKA, HIDEYUKI SHIMAZAKI, TATSUMI KAJI, TOMOHIKO ASANO

**Affiliations:** 1 Departments of Urology, Tokorozawa, Saitama 359-8513, Japan; 2 Hematology, Tokorozawa, Saitama 359-8513, Japan; 3 Basic Pathology, Tokorozawa, Saitama 359-8513, Japan; 4 Radiology, National Defense Medical College, Tokorozawa, Saitama 359-8513, Japan

**Keywords:** mucosa-associated lymphoid tissue lymphoma, upper urinary tract, ureteral wall thickening, nephroureterectomy, rituximab

## Abstract

Mucosa-associated lymphoid tissue (MALT) lymphoma occurs in various sites, but rarely in the urinary tract. Imaging studies of a 69-year-old male revealed a left hydronephrosis and diffuse thickening of the renal pelvic and upper ureteral wall. Retrograde pyelography revealed a narrowing in this region, and brush cytology specimens contained atypical cells. As the lesion was considered to be malignant, surgical excision was performed. Histological analysis revealed an intense lymphoid infiltrate mainly consisting of B cells. The immunohistochemistry results demonstrated that the lesion was positive for CD20 and CD79a and negative for CD5 and cyclin D1. These findings led to a diagnosis of MALT lymphoma. Pathological exploration disclosed a focally dense invasion of lymphoma cells into not only the renal pelvis, but the whole ureteral wall and surrounding tissue; therefore, the patient underwent eight courses of rituximab treatment. Diffuse invasion of lymphoma cells into the whole ureter was rarely observed. Since the surgery, the patient has survived for 78 months without evidence of a recurrence of lymphoma.

## Introduction

Malignant lymphoma of the mucosa-associated lymphoid tissue (MALT) was first reported in 1983. The disease commonly occurs in the stomach and lungs, but rarely in the urinary tract. As MALT lymphoma is one of the less aggressive lymphomas and tends to remain localized with slow progression for a long period, it often produces no subjective symptoms and is incidentally detected in radiological imaging studies. It is occasionally difficult to differentiate MALT lymphoma from urothelial carcinoma using imaging studies when the disease presents with upper urinary obstruction due to renal pelvic or ureteral involvement. In the present study, we report a unique case of MALT lymphoma in which the lymphoma cells appear to diffusely infiltrate the whole upper urinary tract. Written informed consent was obtained from the patient.

## Case report

A 69-year-old male with a history of hypertension and diabetes mellitus was referred to the National Defense Medical College hospital, Tokorozawa, Japan as an abdominal computed tomography (CT) scan had incidentally revealed a left hydronephrosis and abnormal structure surrounding the left renal pelvis.

There were no remarkable findings in the physical examination, while the laboratory tests yielded a white blood cell count of 5.7×10^9^/l (neutrophilic leukocytes, 54.9%), a hemoglobin level of 15.3 g/dl, a platelet count of 247×10^9^/l and a serum creatinine level of 0.92 mg/dl. The patient was free from systemic inflammation; the erythrocyte sedimentation rate (ESR) was 8 mm in the first hour and the C-reactive protein level was <0.3 mg/dl. The urinalysis was normal and the urine cytology indicated no atypical cells.

**Figure 1:**
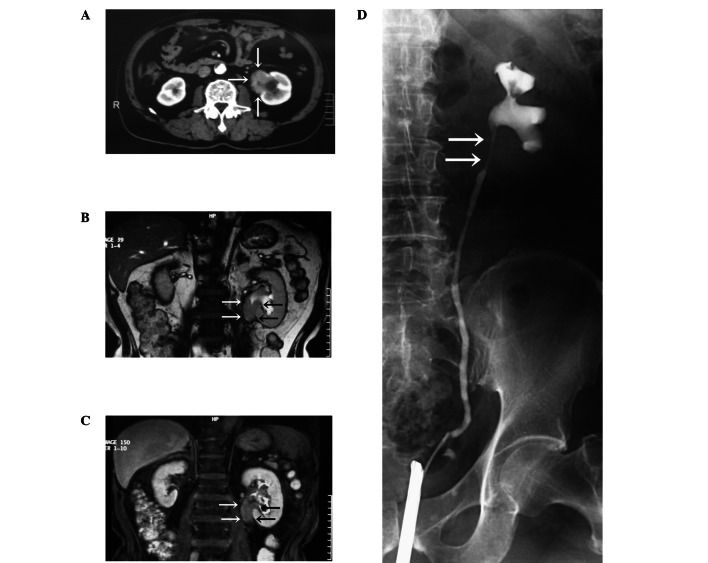
Imaging studies. (A) Abdominal computed tomography (CT) revealing a left hydronephrosis and diffuse pelvic wall thickening (arrows) that are enhanced slightly by contrast media. (B) T2-weighted magnetic resonance imaging (T2WI-MRI) demonstrating a mass-like lesion (arrows) surrounding the renal pelvis and upper ureter, whose signal intensity is slightly lower than that of the renal parenchyma. (C) Gadolinium-enhanced MRI reveals a poorly enhanced pyeloureteral junction with a thickened wall (arrows). (D) Retrograde pyelograpy shows a >3 cm narrowing of the upper ureter (arrows), and the surface of this area appears to be smooth.

Enhanced CT and magnetic resonance imaging (MRI) scans showed a mild left hydronephrosis and a diffuse renal pelvic wall thickening that was enhanced slightly by contrast media (Fig. 1A–C). A left retrograde pyelography revealed a 3-cm long narrowing of the upper ureter and the surface of the ureteral lumen was smooth (Fig. 1D). Brush cytology of the stenotic area revealed atypical cells. Although malignant lymphoma was initially suspected due to the radiological findings, ^67^gallium scintigraphy showed only weak uptake in the left renal pelvis and the CT scan showed no lymph node swelling in the para-aortic area. The serum IL-2 receptor level was slightly elevated (524 U/ml).

**Figure 2:**
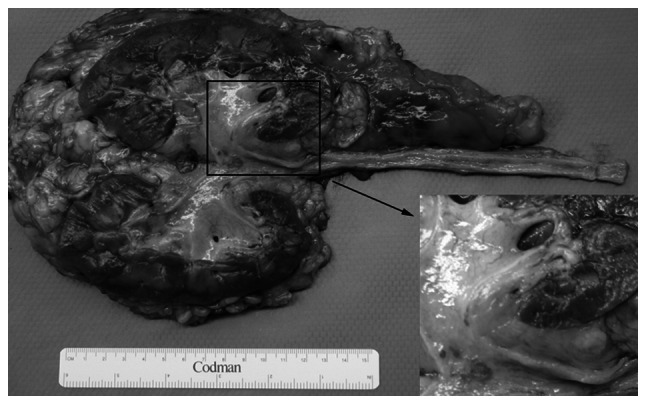
Resected specimen. Macroscopically, a mass lesion surrounding the left renal pelvis and upper ureter presenting as a beige, 10-mm thick, homogeneous, solid component between the urothelial mucosa and para-pelvic fat tissue. The tumor is 4×3×7 cm in size.

The atypical cells identified by brush cytology, and the diffuse thickening of the renal pelvic and ureteral wall detected by imaging studies, led us to suspect invasive urothelial cancer. Therefore, a left nephroureterectomy with bladder-cuff excision was performed. En bloc excision of the left kidney, ureter and para-aortic lymph nodes was conducted, as the malignant lesion was presumed to exist around the renal pelvis and ureter (Fig. 2). Marked adhesion of the left ureter was observed around the common iliac artery, and abnormal fibrotic tissue around the ureter was also resected during the nephroureterectomy.

**Figure 3:**
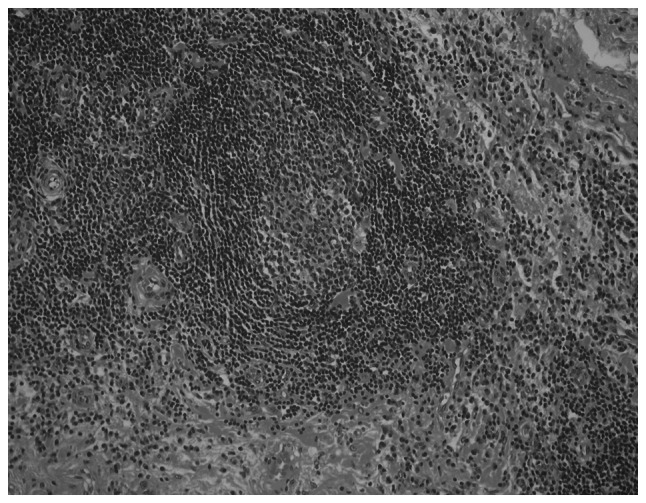
HE staining (×100) showing an intense lymphoid infiltrate mainly consisting of B cells. Certain areas in which plasma cells and small centrocyte-like lymphocytes have formed distinct lymphoid follicles are evident.

Histological examination showed an intense lymphoid infiltrate consisting of mainly B cells. There were areas in which plasma cells and small centrocyte-like lymphocytes formed distinct lymphoid follicles, therefore, extranodal marginal zone B cell MALT-type lymphoma, follicular lymphoma or mantle cell lymphoma were suspected (Fig. 3). The immunohistochemical analysis demonstrated that the lesion was positive for CD20 and CD79a and negative for CD5 and cyclin D1. These observations led to the diagnosis of MALT lymphoma. Lymphoma cells occupying the submucosa of the renal pelvis and upper ureter spread into the surrounding area and extended to the level of the distal ureter, where the abnormal tissue was macroscopically unremarkable. Furthermore, lymphoma cells were identified at the distal edge of the excised ureter. As a focal infiltration of lymphocytes and plasma cells was observed in the specimen obtained from around the common iliac artery, the presence of residual MALT lymphoma *in situ* was inferred. The patient was referred to the department of hematology, where he underwent eight courses of rituximab. Since the surgery, the patient has survived for 78 months without a relapse of lymphoma.

## Discussion

MALT lymphoma, first described by Isaacson *et al* in 1983 (1), accounts for 8% of all malignant lymphomas. The disease may occur in various sites, most commonly in the gastrointestinal tract, lungs, salivary glands, orbits, skin and thyroid, but the urinary tract is rarely involved. Low-grade MALT lymphomas often have an indolent clinical course, and the onset of MALT is usually preceded by an inflammatory process. For example, *Helicobacter pylori* gastritis typically precedes MALT lymphoma of the stomach, lymphoid interstitial pneumonia may precede MALT lymphoma of the lungs, Sjögren syndrome may precede MALT lymphoma of the salivary glands and Hashimoto’s thyroiditis may precede MALT lymphoma of the thyroid. The pathogenesis of MALT lymphoma in the present case is unknown as the patient had no history of urinary tract inflammation and no inflammatory cells in the urine.

To the best of our knowledge, only seven cases of MALT lymphoma affecting the upper urinary tract have been reported previously. Table I summarizes the clinicopathological findings of these cases and those of the present case (2–7). MALT lymphoma of the upper urinary tract is dominant in males (87.5%). The patient age ranged from 30 to 83 years (median, 65 years). Only three patients complained of back or abdominal pain, and the remaining five patients were asymptomatic. Two patients also had cancerous tissue in either the orbits or the salivary and prostate glands. Two of these eight were diagnosed by needle biopsy, and in the case of the remaining six patients, the diagnosis was made after nephrectomy or nephroureterectomy. No cancer-related mortality was reported in these cases, suggesting that patients with MALT lymphoma of the upper urinary tract have a favorable prognosis.

Lymphomatous involvement of the ureter was first reported by Stow in 1909 (8). Ureteral obstruction is rarely observed at the initial presentation of malignant lymphoma, and its overall frequency in malignant lymphoma cases varies from 0.86–8.8% (9–10). Although the majority of patients with lymphomatous involvement of the ureter have extrinsic ureteral compression due to enlarged lymphoma-bearing lymph nodes, upper urinary obstruction due to an apparent intrinsic renal pelvic or ureteral involvement without extrinsic nodal enlargement has rarely been reported. Additionally, diffuse invasion of lymphoma cells into the whole ureter was also rarely observed. In the reported cases listed in Table I, we identified four cases (50%) with similar features of wall thickening; each of these cases presented with a thick urinary tract. Diffuse thickening of the pyeloureteral wall may therefore be a representative feature of MALT lymphoma involving the upper urinary tract.

The overall prognosis for MALT lymphoma patients appears to be good, as MALT lymphoma presents as an indolent and localized disease and remains confined to the site of origin for a prolonged period following diagnosis (11). However, transformation to high-grade lymphoma in the late course of the disease was reported to occur in 8% of MALT lymphoma patients (12). The disease may be treated with surgery or radiotherapy if it is localized, but may require treatment by chemotherapy, with or without irradiation, in cases where it presents with dissemination or high-grade transformation. In the present case, the observation of diffuse lymphomatous extension into not only the renal pelvis but also the whole ureter, and the possibility of residual lymphoma cells around the ureter, led us to administer rituximab following surgery.

## Figures and Tables

**Table I:** Summary of MALT lymphoma affecting the upper urinary tract.

Case	Site	Age (years)	Gender	Chief complaint	Other sites	Radiological feature	Other illness	Means of diagnosis	Additional treatment	Outcome	Ref.
1	Bil. renal pelvis	68	M	None	Salivary and prostate	Mass	Excision of submandibular glands	Biopsy	None	13 months, alive	2
2	Rt renal pelvis and parenchyma	50	M	None		Mass	H.P. gastritis and HT	Nx	Eradication of H.P	Alive	3
3	Rt renal pelvis and parenchyma	30	M	Rt. abd. pain and frequency		Mass	Nephrotic syndrome	Nx	None	28 months, alive	4
4	Lt renal pelvis	83	F	Back pain		Thickening	None	Biopsy	Chemotherapy	8 months, alive	5
5	Rt renal pelvis	72	M	Abd. pain and fever	Bil. orbit	Mass	Colon AC	Nx	None	Died of pulmonary embolism	5
6	Rt renal pelvis	77	M	None		Thickening	Gastric AC	Nux	None	10 months, alive	6
7	Rt upper ureter	72	M	None		Thickening	DM	Nux	None	9 months, alive	7
8	Lt renal pelvis and upper ureter	69	M	None		Thickening	HT and DM	Nux	Chemotherapy	78 months, alive	Present case

MALT, mucosa-associated lymphoid tissue; Bil., bilateral; Rt, right; Lt, left; Abd., abdominal; H.P., *Helicobacter pylori*; HT, hypertension; AC, adenocarcinoma; DM, diabetes mellitus; Nx, nephrectomy; Nux, nephroureterectomy.
